# Insights into Diversity, Distribution, and Systematics of Rust Genus *Puccinia*

**DOI:** 10.3390/jof9060639

**Published:** 2023-05-31

**Authors:** Shubhi Avasthi, Ajay Kumar Gautam, Mekala Niranjan, Rajnish Kumar Verma, Samantha C. Karunarathna, Ashwani Kumar, Nakarin Suwannarach

**Affiliations:** 1 School of Studies in Botany, Jiwaji University, Gwalior 474011, India; shubh.avasth@gmail.com; 2 School of Agriculture, Abhilashi University, Mandi 175028, India; 3 Patanjali Herbal Research Department, Patanjali Research Institute, Haridwar 249405, India; dr.ashwanikumar@patanjali.res.in; 4 Department of Botany, Rajiv Gandhi University, Rono Hills, Doimukh, Itanagar 791112, India; neeru436@gmail.com; 5 Fungal Biotechnology Lab, Department of Biotechnology, School of Life Sciences, Pondicherry University, Kalapet 605014, India; 6 Department of Plant Pathology, Punjab Agricultural University, Ludhiana 141004, India; vermarajnish1985@gmail.com; 7 Center for Yunnan Plateau Biological Resources Protection and Utilization, College of Biological Resource and Food Engineering, Qujing Normal University, Qujing 655011, China; samanthakarunarathna@gmail.com; 8 National Institute of Fundamental Studies (NIFS), Hantana Road, Kandy 20000, Sri Lanka; 9 Research Center of Microbial Diversity and Sustainable Utilization, Chiang Mai University, Chiang Mai 50200, Thailand; 10 Department of Biology, Faculty of Science, Chiang Mai University, Chiang Mai 50200, Thailand

**Keywords:** current status, ITS, LSU, phylogeny, *Pucciniomycotina*, rust fungi, taxonomy

## Abstract

*Puccinia*, which comprises 4000 species, is the largest genus of rust fungi and one of the destructive plant pathogenic rust genera that are reported to infect both agricultural and nonagricultural plants with severe illnesses. The presence of bi-celled teliospores is one of the major features of these rust fungi that differentiated them from *Uromyces*, which is another largest genus of rust fungi. In the present study, an overview of the current knowledge on the general taxonomy and ecology of the rust genus *Puccinia* is presented. The status of the molecular identification of this genus along with updated species numbers and their current statuses in the 21st century are also presented, in addition to their threats to both agricultural and nonagricultural plants. Furthermore, a phylogenetic analysis based on ITS and LSU DNA sequence data available in GenBank and the published literature was performed to examine the intergeneric relationships of *Puccinia*. The obtained results revealed the worldwide distribution of *Puccinia*. Compared with other nations, a reasonable increase in research publications over the current century was demonstrated in Asian countries. The plant families *Asteraceae* and *Poaceae* were observed as the most infected in the 21st century. The phylogenetic studies of the LSU and ITS sequence data revealed the polyphyletic nature of *Puccinia.* In addition, the presences of too short, too lengthy, and incomplete sequences in the NCBI database demonstrate the need for extensive DNA-based analyses for a better understanding of the taxonomic placement of *Puccinia*.

## 1. Introduction

*Puccinia* Pers. is an obligatory plant pathogenic genus of rust fungi that belongs to the family *Pucciniaceae* of the order *Pucciniales* (*Basidiomycota*). It is the largest genus of rust fungi, containing about 4000 species [1,2,3,4,5,6,7], and it has a worldwide distribution. The recently published manuscript “The Outline of Fungi” provides a total of 3300 accepted species of *Puccinia* [8]. This genus of rust fungi is reported to infect a variety of plant hosts found in all land areas except the polar regions. Although various species of *Puccinia* parasitize large groups of vascular plants, the members of the plant families *Asteraceae*, *Cyperaceae*, *Fabaceae*, *Liliaceae*, *Malvaceae*, and *Poaceae* serve as hosts for a large number of them. A rust genus is a group of plant pathogenic fungi that are responsible for serious diseases in both agricultural (e.g., wheat, barley, and oats) and nonagricultural crops (e.g., *Cynodon* sp., *Fagopyrum* sp., *Grewia* sp., *Parthenium* sp., and *Rubia* sp.) [1]. Because of the recognition and importance of the species of *Puccinia* as global rust pathogens, this rust genus has a well-defined history. These rust pathogens have been reported to cause several globally important plant diseases, such as black stem rust and leaf brown rust of small grains and other grasses, stripe or yellow rust of wheat and other grasses, crown rusts of oats and other grasses and rust of common corn, sugarcane, sunflower, safflower, cotton, asparagus, mint, snapdragon, hollyhock, and many more. Due to the involvement of humans everywhere, their activities, along with other physical and biological agents, may promote the global spread of many rust fungi from unknown centers of origin.

The species of *Puccinia* often cause severe losses especially in cereals and gramineous crops across the globe. These are obligate parasites that spread through spores and infect the aerial parts of the host. This spread and further infection are sometimes complete on a single host, or another host is required to complete the life cycle of the rust fungus. Therefore, in nature, some species of *Puccinia* are autoecious (the life cycle is completed on a single species of the host), while others are heteroecious (two host plant species are required to complete the life cycle). The life cycle of *Puccinia* spp. is more complex, compared with those of other plant pathogenic fungi because they have different spore stages (up to five) infecting single or two taxonomically different plant hosts. A maximum of five spore types can be produced by these fungi, depending on the species, environment, and season. The fungi start infection with the formation of asexual urediniospores on the main host (primary infection), which further infect the neighboring plants (secondary infection) of the same host plant. The infection by these urediniospores generally occurs during summer, while sexual teliospores are normally produced near the end of the season and overwinter on plant debris. The teliospores then germinate in the spring season and produce basidiospores, which ultimately disseminate all over again and start infecting the secondary or alternate host (for heteroecious rusts), or start the infection of the same host (for autoecious rusts). Here, the spore types, namely, pycniospores, are produced within pycnia, and, later, aeciospores are generally produced and complete the life cycles of these fungi. However, the produced aeciospores are dispersed by different dispersal agents and infect the primary host once again. Some species of *Puccinia* form all five spore types, which are known as macrocyclic species, while species that lack urediniospores are demicyclic, species that lack pycniospores and aeciospores are hemicyclic, and species that lack pycniospores, aeciospores, and urediniospores are microcyclic [1,9,10].

*Puccinia* species infect nearly every category of plants; however, the species that cause rusts on cereals are the most economically important. Many serious diseases are caused by the species of *Puccinia* (e.g., *Puccinia coronata* infects mainly oats; *P. graminis* infects mainly wheat, barley, and oats; *P. helianthi* infects sunflower; *P. hordei* infects barley; *P. purpurea* infects sorghum; *P. melanocephala* infects sugarcane; *P. recondita* infects mainly rye; *P. sorghi* infects maize; *P. striiformis* infects mainly wheat and barley; *P. triticina* infects wheat; and *P. malvacearum* infects hollyhock). Of all the wheat rust diseases, *Puccinia graminis*, *P. triticina*, and *P. striiformis* cause the rust of wheat, barley, and rye stem, leaves, and grains, primarily occurring in most wheat-growing areas all over the world. They cause severe seasonal diseases in India, and they generate serious outbreaks in North America, Mexico, and South America. *Puccinia graminis*, the original species of *Puccinia*, was examined as a biological warfare agent during the Cold War in addition to being studied as a plant pathogen [11]. The present overview sheds light on the current status of the genus *Puccinia*, with a special reference to up-to-date information on the numbers of species, trends in the last decade, asexual and sexual states, and molecular studies. Other aspects based on diversity and distribution, are also discussed to provide an understanding of the complete distributional range of these fungi.

## 2. The Genus *Puccinia*: General Taxonomy and Ecology

Kingdom: *Fungi*
Division: *Basidiomycota*
Class: *Pucciniomycetes*
Order: *Pucciniales*
Family: *Pucciniaceae*
Genus: *Puccinia* Pers. (1801)
Type species: *Puccinia graminis* Pers. (1794)

Rusts are obligate parasites that show phenotypic and genetic plasticity because of their complete dependency on the presence of living host plants to complete their life cycles [1,12]. These fungi show unique systematic characteristics by producing interesting morphologically and cytologically distinct spore-producing structures, which have attracted the interest of mycologists for centuries. The species of *Puccinia* may produce up to five morphologically and cytologically distinct spore-producing structures. The production of these distinctive structures differentiates these rust fungi from other fungal groups. These structures are produced by the species of *Puccinia* in the infection process of the host-pathogen interaction. These diverse structures are generally the successive stages of reproduction produced by rust fungi, and they may vary from species to species. These fungi commonly appear as yellow-orange or brown pustules on healthy and vigorously growing plant parts, such as leaves, petioles, tender shoots, stems, and fruits. The infection pustules are often associated with chlorotic lesions, which may cause the premature wilt and senesce of infected leaves in cases of severe infection. The spore-producing pustules are present as solitary, scattered, or aggregated in groups, arranged linearly, concentrically, or irregularly, and are often erumpent. The spore-producing structures of the species of *Puccinia* are the basic spore states, which are generally recognized as spermogonium, aecium, uredinium, telium, and basidium. These states are generally assigned Roman numerals (0, I, II, III, and IV, respectively) during the taxonomic description of rust fungi, including *Puccinia* spp. Only a few species of *Puccinia*, such as *P. vexans* Farl., produce six morphologically and functionally different spore stages. Besides the production of regular pale-colored urediniospores, this species produces thick-walled dark-pigmented urediniospores called amphispores. Two different systems are generally suggested, morphology and ontogeny, and they have been applied in the definition and terminology of the spore states of rust. It is important to discuss this aspect here because the terminology used to describe the biology of *Puccinia* spp., including the morphology and life cycle, are also the same as that used for all rust fungi. The spore morphology is generally considered the basis for defining the spore states [13,14]. The spore definitions based on this system are as follows:

Aeciospores: defined as being produced in chains and with ornamentation that is traditionally known as verrucose;

Urediniospores: defined as being always unicellular and borne singly on pedicels, and usually with ornamentation that is traditionally known as echinulate [15].

In the ontogenic system, the position of the spore states in the life cycle rather than the morphology is utilized for defining the spore terminology [16,17,18,19]. The general descriptions of these diverse spores as they are produced by rust fungi (including *Puccinia* spp.) are as follows:

Teliospores: spores that produce basidia (probasidia and hypobasidia);

Basidiospores: spores produced on basidia and are haploid and frequently binucleate, but that are not dikaryotic spores;

Spermatia: dikaryotizing elements;

Aeciospores: dikaryotic nonrepeating spores that are produced in sori typically associated with spermogonia, and that give birth to dikaryotic vegetative mycelia;

Urediniospores: repeated dikaryotic mycelia that typically give rise to dikaryotic mycelia on the same host, and that are sometimes referred to as uredospores or urediospores.

The introduction of a taxonomic grouping as *Forma specialis* (plural *formae speciales*) is allowed by the International Code of Botanical Nomenclature (ICBN). In the case of fungi, it is applied a taxonomic grouping. It is generally adapted when authors do not feel a subspecies or variety name is appropriate. For example, *Puccinia striiformis* Westend. consists of several formae speciales based on host specialization, including *P. striiformis* f.sp. *tritici*, *P. striiformis* f.sp. *hordei*, *P. striiformis* f.sp. *elymi*, *P. striiformis* f.sp. *agropyri*, and *P. striiformis* f.sp. *secalis*. Among the five forms of *P. striiformis*, the sexual stage was confirmed only for the wheat form of the rust *P. striiformis* f.sp. *tritici*, but not known for the other four forms. Based on the host specificity, the numbers of *Forma specialis* of *Puccinia graminis* such as *P. graminis* f.sp. *avenae*, *P. graminis* f.sp. *secalis*, and *P. graminis* f.sp. *tritici*,. are available, which were proved to be helpful in the taxonomy of rust fungi including the genus *Puccinia* [1,2,3].

**Figure 1:**
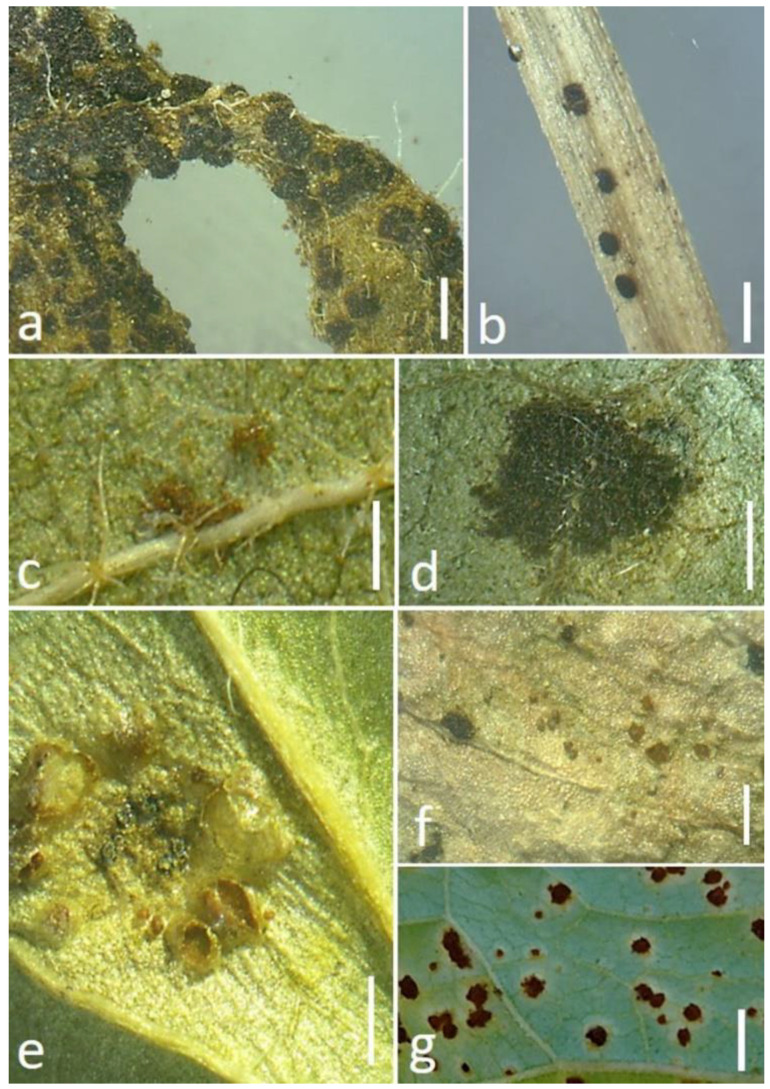
Occurrences of teliosori of different *Puccinia* species with some host plants: (**a**) *Puccinia abrupta* var. *partheniicola* on *Parthenium* sp.; (**b**) *Puccinia cynodontis* on *Cynodon dactylon*; (**c**) *Puccinia tiliaefolia* on *Grewia tiliifolia*; (**d**) *Puccinia himachalensis* on *Clematis* sp.; (**e**) *Puccinia clematidis* on *Clematis* sp.; (**f**) *Puccinia colletiana* on *Rubia* sp.; and (**g**) *Puccinia fagopyri* on *Fagopyrum esculentum*. Scale bar = 1 mm. (Photo taken by Dr. Ajay Kumar Gautam).

**Figure 2:**
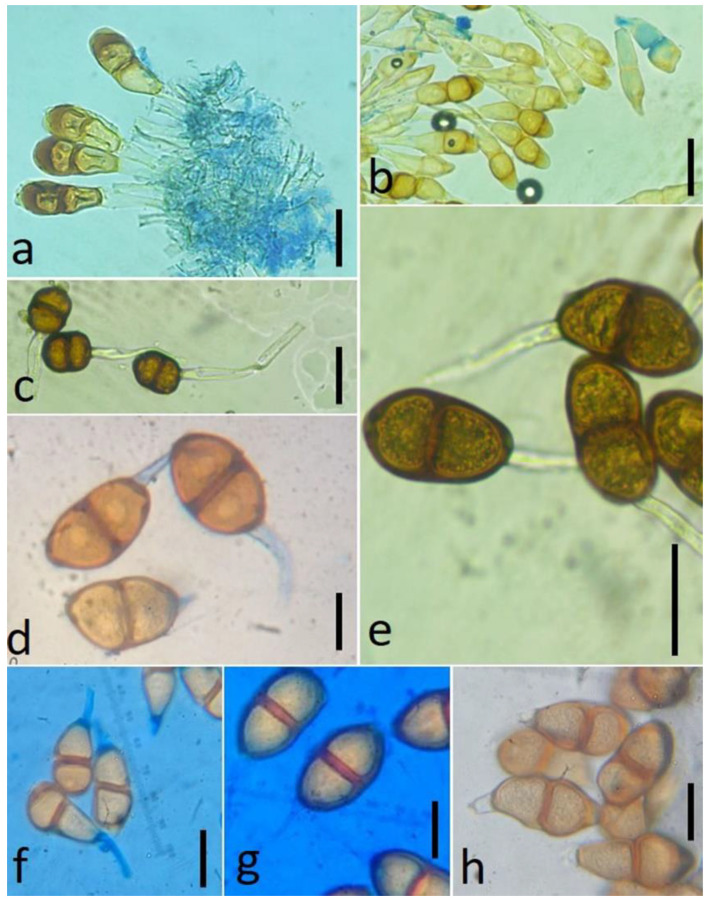
Teliospores of different *Puccinia* species: (**a**) *Puccinia colletiana* from *Rubia* sp.; (**b**) *Puccinia cynodontis* from *Cynodon dactylon*; (**c**) *Puccinia abrupta* var. *partheniicola* from *Parthenium* sp.; (**d**) *Puccinia clematidis* from *Clematis* sp.; (**e**) *Puccinia himachalensis* from *Clematis* sp.; (**f**) *Puccinia tiliaefolia* from *Grewia tiliifolia*; (**g**) *Puccinia himachalensis* from *Clematis grata*; and (**h**) *Puccinia fagopyri* from *Fagopyrum esculentum*. Scale bar = 10 µm. (Photo taken by Dr. Ajay Kumar Gautam).

It is believed that the rust pustules of uredinia that are present on the stem and leaf sheath tissues often survive for a longer duration in comparison with those that are present on the leaves. In the case of spore production, the number of spores produced by leaf pustules is generally higher. Under continuous conditions, stem rust urediniospores show more resistance to atmospheric conditions than leaf rust spores [20,21]. The species of *Puccinia* are responsible for causing all possible types of rust disease symptoms by producing all five basic spore states, which are generally recognized as spermogonium, aecium, uredinium, telium, and basidium. There is great variation in the production of the spore states by the different species of this rust genus. While some species produce all the spore states, others may produce less than five. The teliosori and teliospores of different *Puccinia* species are presented in Figure 1 and Figure 2, respectively.

## 3. Data Collection and Compilation

This paper was compiled based on the information retrieved from an extensive search of peer-reviewed publications, field guides, monographs, books, conference proceedings, project reports, dissertations, theses, and other offline and online resources. Information based on taxonomic studies, checklists, and new reports, as well as reports on the new taxa, was generally considered in the compilation of this study. The scientific names of the hosts and fungi were then cross-verified for scientific entities. The Plant List (http://www.theplantlist.org, accessed on 20 April 2022) was consulted for the verification of the host plant names, and the fungal databases MycoBank (www.mycobank.org; accessed on 20 April 2022), Species Fungorum (www.speciesfungorum.org; accessed on 20 April 2022), and IndexFungorum (www.indexufngorum.org; accessed on 20 April 2022) were consulted to gather information on the current fungal names, numbers, and distributions. Fungal Databases, US National Fungus Collections, ARS, and USDA, which are important online sources of plant pathogens and their hosts, were also used during the compilation. To understand the general trend of the outline and a higher-rank classification of *Puccinia*, publications such as [1,4,5,6,8,22,23] were consulted. An attempt was made to summarize all the collected information in the form of the current statuses of the species numbers, their distributions with respect to hosts, and the trends of the published literature in the last century and decade. The publication indices of *Puccinia* spp. in terms of year, decade, and century were also analyzed and are presented in this paper. In addition, the references in other languages were translated into English so that the scientific community could easily understand them. In addition, the role of *Puccinia* as a threat to biodiversity is also discussed on a global scale in the present paper. A short discussion on the limitations to the current knowledge of *Puccina* and future recommendations is also presented here.

## 4. Phylogenetic Analyses

We worked on the *Puccinia* species phylogeny, and the NCBI search showed 292,000 *Puccinia* hits, of which most were repeated and whole-genome sequences. An NCBI search with “*Puccinia* and type” showed 52,446 sequence results, of which 13 sequences were found to be type sequences. We chose Index Fungorum to search for species deposited more than two decades between 2000 and 2022 (till July), for which a total of 228 species were found. From the above two sources, we collected the ITS (69), LSU (65), SSU (15), cytochrome oxidase *COX* (9), *TUB* (8), *RPB2* (1), and *TEF1* sequences. Because most regions have a small number of sequences, we selected the ITS and LSU sequences to construct the multigene phylogeny. The DNA sequence data of the *Puccinia* species from the LSU and ITS rDNA were downloaded from GenBank and earlier published literature. Individual nucleotide sequences of the LSU and ITS were distinctly aligned using the MAFFT v7.450 online server (https://mafft.cbrc.jp/alignment/server/; accessed on 20 April 2022) and exported to aligned sequence data [24], and they were then manually checked and edited where necessary in BioEdit v.7.0.9 [25]. The sequences of taxa containing weakly aligned portions, incomplete data, missing sequence data, and gaps were removed. The separate aligned gene regions of the LSU and ITS were combined in BioEdit. The combined multigene sequence alignment was converted to the PHYLIP format (.phy) using the alignment transformation environment (http://sing.ei.uvigo.es/ALTER/; accessed on 20 April 2022) for randomized accelerated maximum likelihood (RAxML) analysis. The aligned LSU and ITS single-gene datasets and a concatenated dataset of LSU and ITS genes were analyzed with maximum likelihood using the RAxML-HPC2 on XSEDE (8.2.8) [26,27] on the CIPRES Science Gateway platform [28] using the GTR + I + G model of evolution. Maximum likelihood bootstrap values equal to or greater than 70% were given above each node. Phylogenetic trees were visualized with the FigTree v.1.4.0 program [29] and reorganized in Microsoft PowerPoint. A checklist of molecular studies on *Puccinia* spp., along with the names of the isolates, was also prepared and is presented in Table 1.

**Figure 3:**
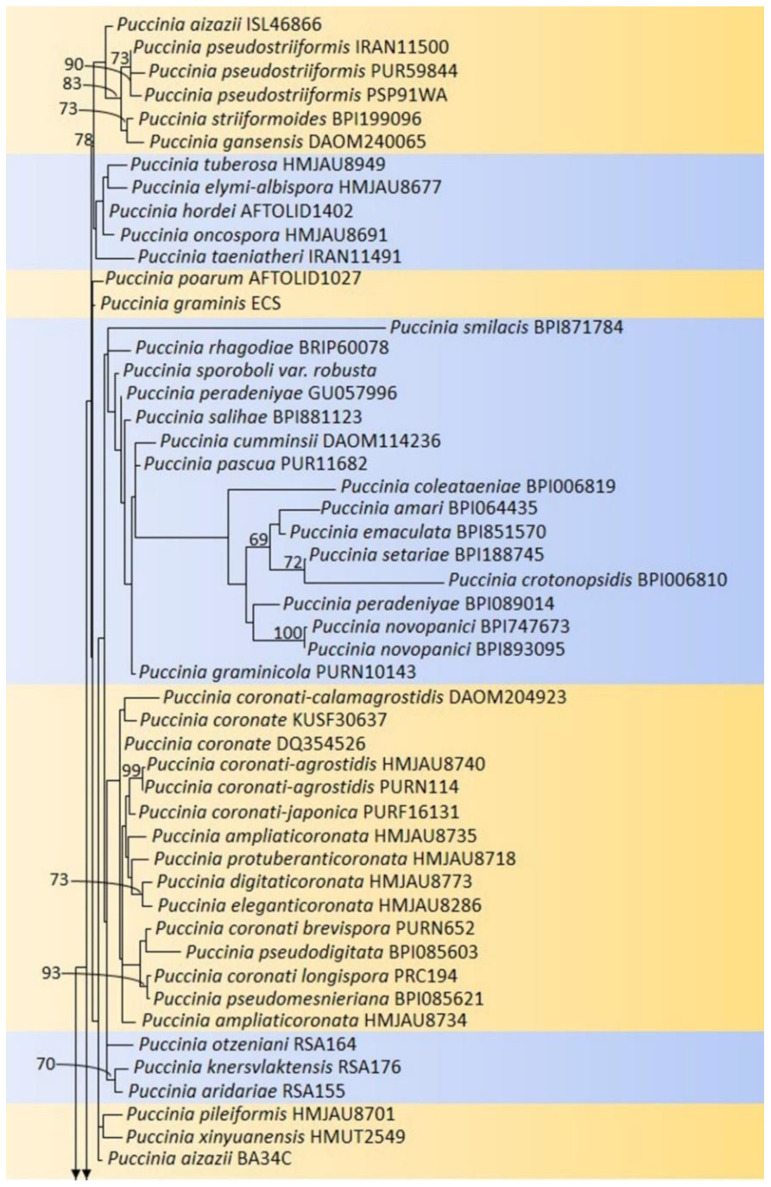

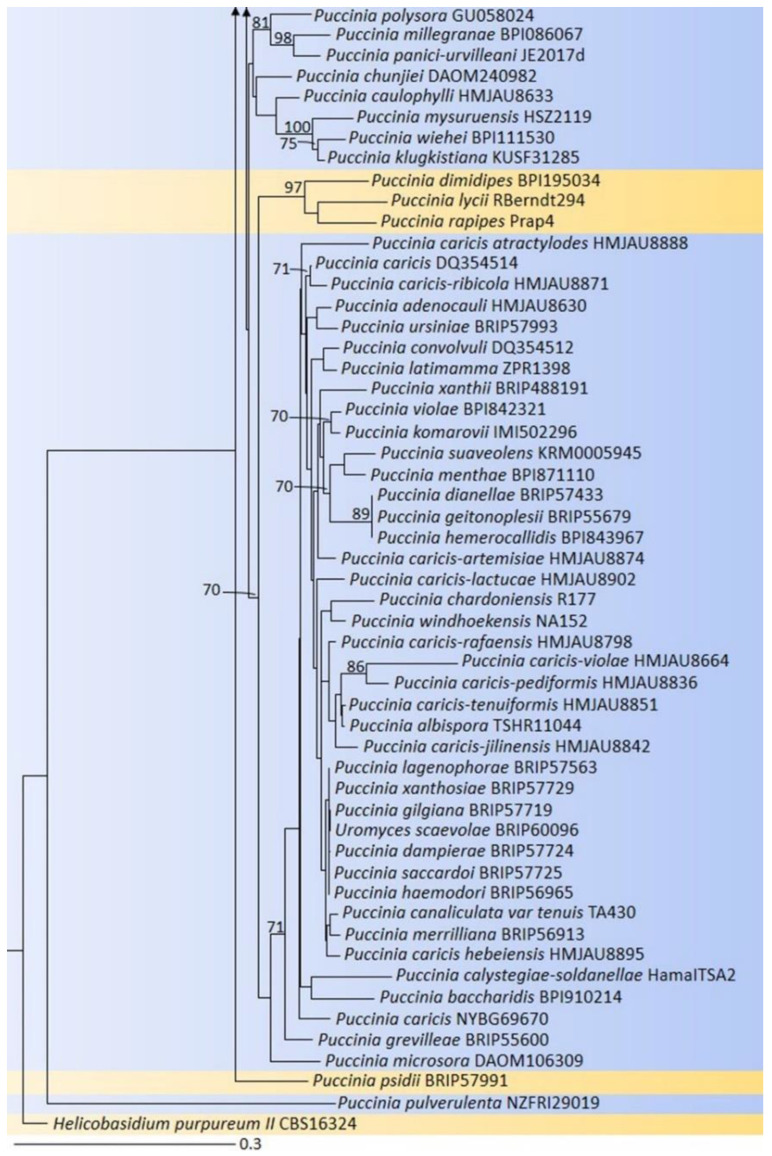
Multigene phylogeny of *Puccinia* species. Maximum likelihood tree alignment of 134 reference ITS and LSU sequences along with outgroup. Alignment was performed with MAFFT v.7.450 online server (https://mafft.cbrc.jp/alignment/server/; accessed on 20 April 2022) exported to aligned sequence data. Sequences were edited in Change the FASTA to PHYLIP format (http://www.sing-group.org/ALTER/; accessed on 20 April 2022). The RAxML-HPC2 in XSEDE (version 8.2.8) [27] on the CIPRES Science Gateway platform [28] was used with the GTR + I + G evolution model. The phylograms were generated by FigTree v.1.4.0 [29] and were reorganized in Microsoft PowerPoint. Tree nodes represent ≥70% bootstrap values. Scale bar represents number of substitutions expected per site. The tree is rooted with *Helicobasidium purpureum* CBS 163.24. GenBank accession numbers are listed in Table 1. RAxML analysis yielded a minimum scoring tree with a final ML optimization likelihood value of −12,532.792474. The matrix had 834 distinct alignment patterns, with 47.11% indeterminate characters or gaps. The estimated base frequencies were as follows: A = 0.315347; C = 0.157275; G = 0.229122; T = 0.298256; substitution rate AC = 1.494199; AG = 2.855095; AT = 1.851749; CG = 0.513805; CT = 4.845757; and GT = 1.000000. Proportion of invariable sites: I = 0.127613; and gamma distribution shape parameter: α = 0.463021.

Most of the *Puccinia* species were identified based on the morphology and microscopic characteristics of the uredia and telia, or based on other successive stages observed on the collected samples. The identification of this rust genus based on molecular parameters is not up to the mark and still requires extensive studies. In the phylogenetic results, the *Puccinia* species were separated into two complexes in both the ITS and LSU sequence data. Both complexes of the ITS and LSU share many similar sequences. The incomplete sequences were mostly found in the *Puccinia* sequence dataset (e.g., ITS1 and 5.8S or ITS1, 5.8S complete, and ITS partial or 28S partial). Approximately, 50% of the sequences had up to 300 nucleotides, while the remaining sequences had up to 800 nucleotides. Incomplete sequences can result in two complexes in a single genus. Therefore, complete gene sequences from the ITS and LSU are needed to analyze these complex clades. The phylogenetic analyses exposed the polyphyletic nature of this genus, which requires further DNA-based analyses of the rust disease caused by *Puccinia* to develop a better understanding of its taxonomic placement. The genus *Uromyces* also showed a polyphyletic nature during a study carried out by the authors of [30]. A study carried out by the authors of [6,31,32] also confirmed the polyphyletic nature of the rust genus *Puccinia* (Figure 3).

## 5. Current Status of Numbers of Species

**Figure 4:**
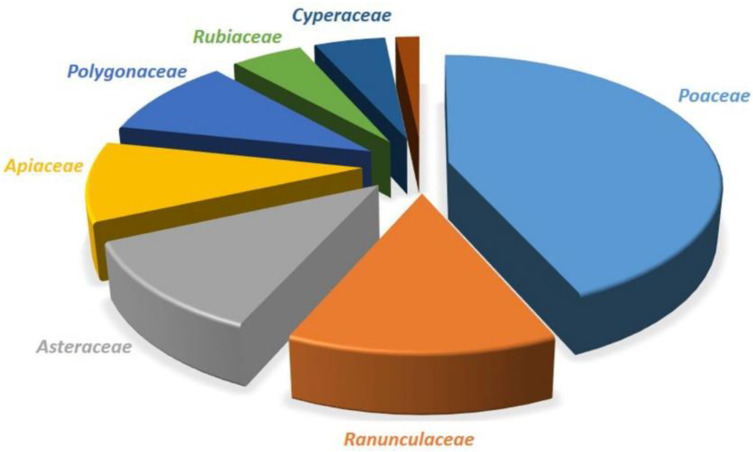
General distribution of *Puccinia* rust on plant host families (based on literature).

The occurrence of the rust genus *Puccinia* is considered cosmopolitan. All the continents, except Antarctica, show the presence of many species of the rust genus *Puccinia*. The genus is one of the broadly studied rust genera, and the fungi of this genus also possess broad host ranges and distributions. Nearly all categories of plants belonging to approximately all the families have been found to be infected with these fungi. Similar to the trends of the occurrences of different rust fungi on their hosts, the occurrence of *Puccinia* rust has also been reported to be the most profuse on plant hosts that belong to the families *Asteraceae*, *Poaceae*, and *Ranunculaceae*. When we came across the number of research papers previously published by several researchers, a similar trend of the occurrence of *Puccinia* rust was observed. Similarly, plant families such as *Apiaceae*, *Polygonaceae*, *Rubiaceae*, *Cyperaceae*, *Acanthaceae*, *Berberidaceae*, *Lamiaceae*, and *Saxifragaceae* are among the most infected plant families with *Puccinia* rust; however, the infection and host range of *Puccinia* rust is not only limited to these plant families. A tentative distribution of *Puccinia* rust in the major plant families is summarized and presented in Figure 4. When we talk about the species boundaries of *Puccinia* rust, a total of 5450 epithets are available on Index Fungorum (www.indexfungorum.com; accessed on 20 April 2022). However, a total of 3300 species of *Puccinia* have been reported all over the world on a variety of hosts [1,2,3,4,5,6,7,8,22,33].

## 6. Trends in Published Literature

In this section, the research on *Puccinia* rust reported and published in various journals is presented. A total of 988 papers were published from the year 1794 to 2020, and they were included in the present study to understand the general publication trend. The data from different online platforms, as well as offline resources, were retrieved to compile the information on these rust fungi. To understand the decadal trend, the numbers of publications were counted per ten years and are presented according to century and some more criteria. Publications on the new records, reports, and taxa (genus/species) were generally included in this analysis and are presented in Table 2.

The results revealed that the research on *Puccinia* rust was quite encouraging during the 19th and 20th centuries. A total of 277 papers were published during the 19th century, while 627 were published during the 20th century. The trend of research publications for the current century (up to 2020) has also been high in terms of both qualitative and quantitative aspects. Similarly, in the decadal analysis of the published research on *Puccinia* rust, the highest numbers of papers were published at the end of the 19th century and the beginning of the 20th century (121 and 144, respectively). A further trend in the decadal analysis of the number of publications on *Puccinia* rust during the 19th century was observed in the following order: 1891–1900 (121), 1871–1880 (61), and 1881–1890 (50). There were less than ten publications in each of the other decades. Similarly, in the 20th century, the trend of publication was as follows: 1901–1910 (144); 1911–1920 (85); 1951–1960 (78); 1931–1940 (71); 1941–1950 (58); 1931–1940 (51); 1971–1980 (44); 1981–1990 (39); 1991–2000 (29); and 1961–1970 (28).

Morphotaxonomy alone is not enough to describe a new taxon. The molecular aspects play an important role in resolving the correct taxonomic placement of all fungi, including rust fungi. This might explain the variations in the number of publications during the current century. Not all researchers can afford a good laboratory and access to resources. Nowadays, insufficient funding is also a major constraint in the performance of basic taxonomic research.

## 7. Threat to Biodiversity

Rust fungi are considered one of the most serious threats to both agricultural (e.g., wheat, soybean, or coffee), and non-agricultural crops and tree species as well. *Puccinia* is one of the harmful biotrophic fungal genera that seriously harm major cereal crops (except rice) and nonagricultural plants all over the world. The type species of *Puccinia* (*Puccinia graminis*) is one of the destructive rust fungi reported to cause the mass destruction of wheat and barley stem rust (black rust). Similarly, *Puccinia striiformis* f. sp. *tritici*, wheat stripe rust (yellow rust), and *P. triticina*, wheat leaf rust (brown rust), are also destructive rusts that are distributed all around the globe. To understand why the species of *Puccinia* are a threat to biodiversity, some examples of rust diseases caused by them are presented in this section.

***Puccinia graminis*** Pers., *Neues Mag. Bot.* 1: 119 (1794)

This is a macrocyclic heteroecious rust that has devastated wheat for many decades, and it is one of the most studied rust fungi. It causes the black stem rust of wheat and poses a serious threat to food security. It may cause crop losses of up to 70%. The *Berberis*, *Berberis*, *Mahoberberis*, and *Mahonia* serve as alternate hosts for this fungus. This fungus occurs in all major wheat-growing areas around the world. *Puccinia graminis* has also been studied in detail and has long been used as a model for studying the cytology, physiology, biochemistry, and molecular aspects of rust fungus biology [34,35,36]. Another extremely contagious race of *Puccinia graminis*, TTKSK (Ug99), was discovered in Uganda. Because it does not recognize any national borders and can infect fields anywhere, it poses a serious threat to wheat growers all over the world. This strain is aggressive against numerous resistance genes that have previously shielded wheat against stem rust. It can cause losses of the victim crop of up to 100%. Although there are Ug99-resistant wheat variants available, their cultivation range is not broad [37,38,39,40].

***Puccinia striiformis*** Westend., *Bull. Acad. R. Sci. Belg.*, *Cl. Sci.* 21 (no. 2): 235 (1854)

This is a biotrophic and heteroecious rust pathogen that has been reported to cause yellow (stripe) rust. At least two lineages are exclusive to grasses, while one lineage primarily infects cereals. The pathogen has the widest host range within the tribe *Triticeae* (in the plant genera *Aegilops*, *Elymus*, *Hordeum*, and *Triticum*) and *Berberis* spp. as the alternate host or sexual host. It is reported to be one of the most damaging cereal rusts compared with other rusts. This fungus reduces the photosynthetic area and the production of sugars for the plant. As the fungus mainly infects the leaf, it also causes substantial water loss while erupting the epidermis. Based on the disease severity and susceptibility of the variety, this rust can cause mild to very high losses; however, a 30% loss is common in susceptible varieties [41].

***Puccinia coronata*** Corda, *Icon. Fung. (Prague)* 1: 6 (1837)

This rust is reported to cause crown rust disease in cultivated and wild oat (*Avena* spp.). It infects two hosts to complete its life cycle: oat (asexual phase) and *Rhamnus* spp. (sexual phase) as the primary and alternate hosts, respectively. Oat crop cultivation areas with warm temperatures (20–25 °C) and high humidity are more prone to this rust epidemic. Infection by the pathogen leads to plant lodging and shriveled grains of poor quality. This rust pathogen can infect 290 species of grass hosts [42,43,44].

***Puccinia psidii*** G. Winter, *Hedwigia* 23: 171 (1884)

The rust *Puccinia psidii* is a pathogen with a broad host range in the myrtle family (*Myrtaceae*). However, the common guava (*Psidium guajava*) and *Eucalyptus* spp. are at more risk, as it causes severe infection in these plants [45,46,47]. A severe infection of *P. psidii* was reported in Brazil, which caused damage to various members of the family *Myrtaceae* [46,48,49]. Similarly, this fungus causes eucalyptus rust in Australia and poses a threat to the biodiversity in this country, as well as to the eucalyptus forest industry worldwide [45]. In 2017, based on a DNA-based molecular analysis of rust samples, the names were synonymized by Beenken in a new genus as *Austropuccinia psidii* [50]. Apart from the abovementioned diseases caused by the rust genus *Puccinia*, these fungi are reported to cause several diseases on other plants. Several research and review papers are available on different online and offline platforms that describe the diversities, distributions, and host ranges of rust fungi, including *Puccinia* [31,32,51,52,53,54,55,56,57,58]. A list of the species of *Puccinia* that cause destructive diseases in agricultural and nonagricultural crops is presented in Table 3.

## 8. *Puccinia* in the Present Century

**Figure 5:**
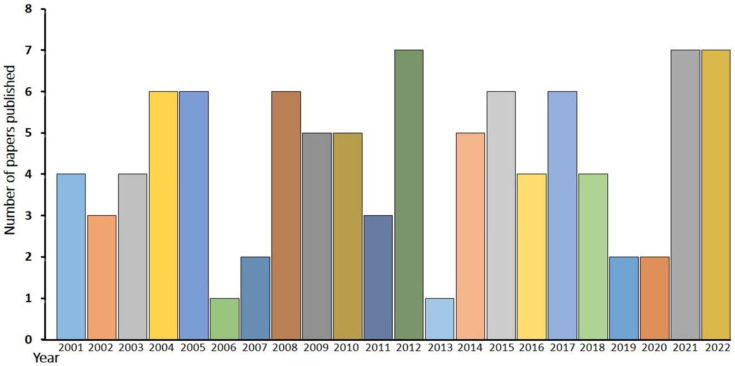
Trends of published literature (by year) on *Puccinia* during the present century.

To understand the status of the rust genus *Puccinia* in the current century, the published literature on new genera and species and new geographical records were taken into consideration, and the data obtained were compiled concerning the host, host family, yearly publication details, and distribution throughout the regions, countries, and continents around the globe. Data based on other aspects of *Puccinia* rust, such as physiology and biochemistry, were not considered in this study. The publication details of the last two decades of the current century reveal that a total of 82 papers on *Puccinia* rust were published, 42 of which were published during 2001–2010, and 40 of which were published from 2010 to the present date (Figure 5).

**Figure 6:**
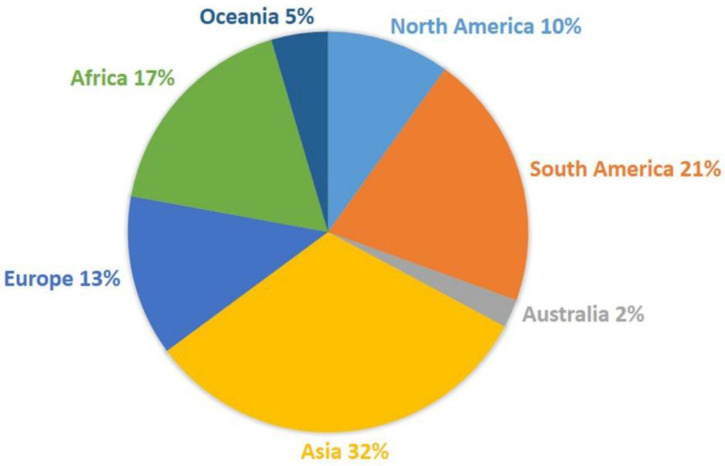
Trends of published literature (by continent) on *Puccinia* during the present century.

After analyzing the yearly data, it was observed that the publication record numbers reached up to seven in many years, while the lowest number (one) was also observed for many years. Further, a total of 126 records in the form of new geographical records from 62 regions belonging to 37 countries were recorded during the last two decades. All seven continents showed distributions of the species of *Puccinia*. If we compare the continental distribution of these rust fungi during the current century, then the highest number of records is found in Asia (42 records from 10 countries), followed by South America (27 records in four countries), Africa (23 records in three countries), Europe (17 records in seven countries), North America (13 records in 11 countries), Oceania (six records in two countries), and Australia (with three records) (Figure 6).

**Figure 7:**
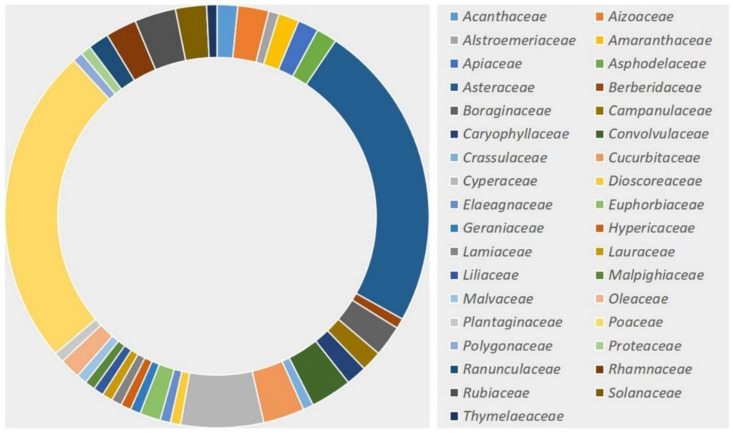
Trends of published literature (by family) on *Puccinia* during the present century.

**Figure 8:**
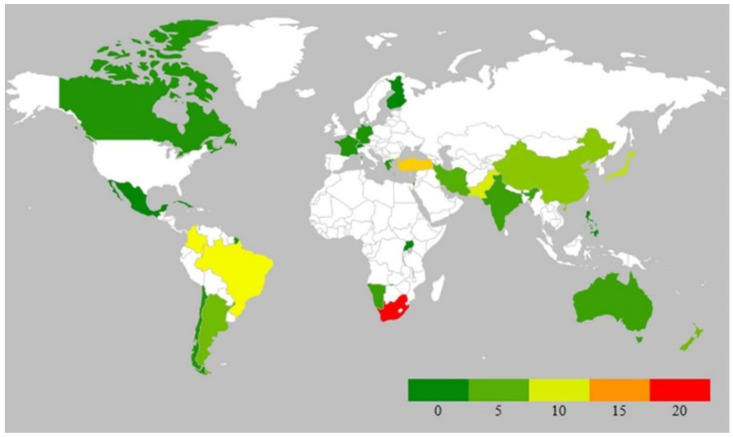
Map showing the occurrence of *Puccinia* rust in the 21st century. Color indicators: green color indicates minimum values (0–5); yellow and orange medium values (10–15); and red color maximum values (15–20).

The rich biodiversity and variable climatic conditions of Asia might be responsible for the occurrence of rust fungi (*Puccinia* spp.) in high numbers. The same explanation is applicable to America and Africa, while the lowest reports from Oceania and Australia directly correlate with the agroclimatic conditions of these two continents. When we analyze all 126 records, only 34 species of *Puccinia* have been identified at the molecular level using multigene analysis. From the data obtained on the host distribution, a total of 124 plant species belonging to 90 plant genera of 34 plant families have been found to be infected with different species of *Puccinia*. As observed in the previous section, *Asteraceae* and *Poaceae* have been found to be highly infected with different species of *Puccinia*. The data on *Puccinia* rust during the present century is summarized and graphically presented in Figure 7 and Figure 8.

## 9. Limitations of Current Knowledge

The current research on rust fungi is mainly based on morpho-taxonomy (the morphologies of the shapes and sizes of certain spore stages), while, the inclusion of recent technologies, and specifically DNA-based techniques, brings a new turn to the taxonomy of rust fungi. When it is not possible to differentiate two similar fungal species based on their morphological characteristics, molecular techniques can be used to successfully differentiate them, even at the difference of one base pair of nucleotides. However, few studies are based on the use of modern instruments and molecular-based techniques for fungal taxonomy, and specifically DNA-based techniques. The fundamental reasons for the slow adoption of molecular techniques in taxonomic studies on the rust fungi of *Puccinia* are the same as those that apply to all other rust fungi. When studying the species of *Puccinia*, mycologists encounter several problems/limitations, including the lack of necessary databases, resources, and funding, the reduced interest of budding researchers, and the pricey services offered by many agencies. To understand these limitations, a detailed explanation is given below:

- Apart from the availability of 5000 species of *Puccinia*, only a few species are known to have DNA sequence data. The difficulty in culturing rust fungi is one of the possible reasons behind the reduced availability of molecular data. Further, DNA isolation directly from rust fungi present on a natural host and further processing are not easy tasks. In addition, the available sequences are not up to the mark. While others are too long, some sequences are too short, and some are incomplete. While some sequences are up to 300 nucleotides long, others are up to 800 nucleotides long, reflecting the intricacy of their taxonomic evaluation. Incomplete sequences in the *Puccinia* sequence dataset can result in two complexes in a single genus. Therefore, to investigate these complicated clades, entire gene sequences are required. The phylogenetic studies revealed the polyphyletic nature of the species of *Puccinia*, which require more DNA-based analyses for a better understanding of their taxonomic placement. The nonavailability of molecular data for all collections of *Puccinia* all over the globe is another limitation that highlights the requirement for fresh collections of *Puccinia* species and their molecular characterizations to generate molecular data so that their phylogenetic relationships can be explained more precisely. Although country-wide databases of rust fungi are available on various online platforms, the lack of a universal platform exclusively for global rust fungi is also a major limitation in the research on rust fungi, including *Puccinia*.
- When we talk about the day-by-day decreased interest of budding scientists/researchers in the field of the basic taxonomy of fungi, the reasons behind this are complex, such as insufficient funds, expensive outsourced mycological services, and, overall, the difficulty in publishing taxonomies in high-impact journals without modern techniques. These issues are leading to the decreased interest of mycologists in fungal taxonomy, which is ultimately decreasing the number of fungal taxonomists [32].
- Publication in high-impact journals has now become a criterion to assess the quality of research and the performance of researchers/scientists/academicians, or to appraise whether they should be promoted. However, taxonomy based on DNA-based molecular techniques has now become a minimum criterion to process any submitted manuscript, even for initial review. Luckily, few journals are still focusing on the novelty of the research, and most are now considering fungal manuscripts that are purely based on morpho-taxonomy.
- Despite being less expensive to support than applied research, basic fungus research is no longer prioritized for funding. This scenario is common in developing countries. The fundamental inventorying and identification of fungi is not everyone’s cup of tea, similar to obtaining funding for applied research (ideally in biotechnology). Due to a lack of sufficient funding, laboratories working on taxonomic studies of fungi continue to lack current equipment (e.g., that which is used in DNA isolation, amplification (PCR), and sequencing), and they are gradually turning their attention to the practical elements of the field. Additionally, not every mycologist can afford the service fees for the molecular techniques offered by many agencies/institutions of national and worldwide reputation, and particularly researchers working on a self-finance basis.

## 10. Conclusions

In conclusion, it was observed that *Puccinia* is the largest genus of rust fungi that infect a wide variety of host plants of both agricultural and nonagricultural importance. The genus shows variation in its diversity and distribution worldwide. Based on compiled data, a total of 5450 epithets (3300 species) are available [70]. In the evaluation of the host distribution, plant families such as *Asteraceae* and *Poaceae* were found to be highly infected with different species of *Puccinia*. The analyses of the trends in the published literature on rust showed that researchers from Asian countries are among those who have published the highest numbers of papers on all the continents. The NCBI search showed 292,000 hits of repeated and whole-genome sequences. While some sequences are too short, others are too lengthy, and some are incomplete. It was also observed that the taxonomic statuses of a number of *Puccinia* spp. are still unclear, as only morpho-taxonomic traits have been used to identify the majority of them. Moreover, molecular data on most *Puccinia* spp. are not available so far, and their taxonomic placement is still doubtful; hence, they are classified as *incertae sedis*. This generates a potential area of research interest for both current and future mycologists. Similarly, the generic names of many *Puccinia* spp. have been changed or transferred to different genera; however, the incorporation of this revision is still required in their original collections (types or records). Furthermore, the phylogenetic analyses exposed the polyphyletic nature of this genus, which requires further DNA-based analyses of the rust disease caused by *Puccinia* to develop a better understanding of its taxonomic placement. A study carried out by the authors of [6,31,32] also confirmed the polyphyletic nature of the rust genus *Puccinia*. Therefore, fresh collections of *Puccinia* species and their molecular characterizations to generate molecular data are highly recommended so that their phylogenetic relationships can be explained more precisely. All these limitations generate excellent opportunities for mycologists to explore this rust genus based on morpho-taxonomy and molecular data to determine and confirm the taxonomic positions of its species. Additionally, the development of a universal digital platform exclusively for global rust fungi is also recommended in the present study so that researchers who are working on this specific group of fungi can take advantage of this information in one place.

## Figures and Tables

**Table 1:** GenBank and voucher/culture collection accession numbers of *Puccinia* species included in phylogenetic analyses.

Fungal Taxa	GenBank Accession Number	
	ITS	LSU
*Helicobasidium purpureum* CBS 163.24	AY292433	AY254181
*Puccinia adenocauli* HMJAU 8630	MK785267	MK785292
*Puccinia aizazii* BA34C	–	KY386659
*Puccinia aizazii* ISL 46866	NR158929	–
*Puccinia albispora* TSHR 11044	MT560796	MT560813
*Puccinia amari* BPI 064435	KX190836	KX190914
*Puccinia ampliaticoronata* HMJAU 8734	–	MW404927
*Puccinia ampliaticoronata* HMJAU 8735	MW404766	–
*Puccinia aridariae* RSA 155	–	DQ917725
*Puccinia baccharidis* BPI 910214	KY764097	–
*Puccinia calystegiae-soldanellae* HamaITSA2	AB125956	–
*Puccinia canaliculata* var. *tenuis* TA 430	–	OL437033
*Puccinia caricis-artemisiae* HMJAU 8874	MW447821	MW414430
*Puccinia caricis-atractylodes* HMJAU 8888	MW447811	MW414419
*Puccinia caricis* BPI 871515	–	DQ354514
*Puccinia caricis-hebeiensis* HMJAU 8895	MW447818	MW414427
*Puccinia caricis-jilinensis* HMJAU 8842	MW447898	MW414367
*Puccinia caricis-lactucae* HMJAU 8902	MW447781	MW414384
*Puccinia caricis* NYBG 69670	MK518859	MK518517
*Puccinia caricis-pediformis* HMJAU 8836	MW447855	MW414323
*Puccinia caricis-rafaensis* HMJAU 8798	MW447892	MW414360
*Puccinia caricis-ribicola* HMJAU 8871	MW447805	MW414413
*Puccinia caricis-tenuiformis* HMJAU 8851	MW447858	MW414326
*Puccinia caricis-violae* HMJAU 8664	MW447798	MW414406
*Puccinia caulophylli* HMJAU 8633	MK785263	MK785288
*Puccinia chardoniensis* R177	–	EU851149
*Puccinia chunjiei* DAOM 240982	NR111548	-
*Puccinia coleataeniae* BPI 006819	KX190843	KX190919
*Puccinia convolvuli* BPI 871465	–	DQ354512
*Puccinia coronata* BPI 844300	–	DQ354526
*Puccinia coronata* KUSF 30637	MT393874	MT393876
*Puccinia coronati-agrostidis* HMJAU 8740	MW404761	MW404921
*Puccinia coronati-agrostidis* PURN 114	NR111528	–
*Puccinia coronati-brevispora* PURN 652	NR111526	–
*Puccinia coronati-calamagrostidis* DAOM 204923	HM131305	–
*Puccinia coronati-japonica* PURF 16131	NR111527	–
*Puccinia coronati-longispora* PRC 194	HM131233	–
*Puccinia crotonopsidis* BPI 006810	KX190844	KX190920
*Puccinia cumminsii* DAOM 114236	KX190845	-
*Puccinia dampierae* BRIP 57724	–	KF690688
*Puccinia dianellae* BRIP 57433	KM249859	–
*Puccinia digitaticoronata* HMJAU 8773	MW404702	MW404862
*Puccinia dimidipes* BPI 195034	MH144395	-
*Puccinia eleganticoronata* HMJAU 8286	MW404721	MW404881
*Puccinia elymi-albispora* HMJAU 8677	MW404820	MW404980
*Puccinia emaculata* BPI 851570	NR148108	–
*Puccinia gansensis* DAOM 240065	HM057115	–
*Puccinia geitonoplesii* BRIP 55679	KM249860	–
*Puccinia gilgiana* BRIP 57719	–	KF690691
*Puccinia graminicola* PURN 10143	MH707050	MH704521
*Puccinia graminis* ECS	–	AF522177
*Puccinia grevilleae* BRIP 55600	–	KX999878
*Puccinia haemodori* BRIP 56965	–	KF690692
*Puccinia hemerocallidis* BPI 843967	DQ354519	–
*Puccinia hordei* AFTOLID 1402	–	DQ354527
*Puccinia klugkistiana* KUSF 31285	MW740211	MW740212
*Puccinia knersvlaktensis* RSA 176	–	DQ917726
*Puccinia komarovii* IMI 502296	KC466553	–
*Puccinia lagenophorae* BRIP 57563	–	KF690696
*Puccinia latimamma* ZPR 1398	MK518986	MK518685
*Puccinia lycii* RBerndt 294	MH144384	–
*Puccinia menthae* BPI 871110	DQ354513	–
*Puccinia merrilliana* BRIP 56913	–	KX999885
*Puccinia microsora* DAOM 106309	MW009501	–
*Puccinia millegranae* BPI 086067	–	NG059683
*Puccinia mysuruensis* HSZ 2119	KC847089	–
*Puccinia novopanici* BPI 747673	NR148109	–
*Puccinia novopanici* BPI 893095	KX190888	KX190947
*Puccinia oncospora* HMJAU 8691	MW404822	MW404982
*Puccinia otzeniani* RSA 164	–	DQ917742
*Puccinia panici urvilleani* JE 2017d	–	BPI841053
*Puccinia pascua* PUR 11682	MH707035	MH704507
*Puccinia peradeniyae* BPI 089014	KX190906	–
*Puccinia peradeniyae* BPI 871072	–	GU057996
*Puccinia pileiformis* HMJAU8701	MW404792	MW404952
*Puccinia poarum* AFTOLID 1027	–	DQ831028
*Puccinia polysora* BPI 863756	–	GU058024
*Puccinia protuberanticoronata* HMJAU 8718	MW404752	MW404912
*Puccinia pseudodigitata* BPI 085603	KF661261	–
*Puccinia pseudomesnieriana* BPI 085621	KF661263	–
*Puccinia pseudostriiformis* IRAN 11500	AY956560	–
*Puccinia pseudostriiformis* PSP91WA	KM507443	–
*Puccinia pseudostriiformis* PUR 59844	MT965634	–
*Puccinia psidii* BRIP 57991	–	KF318443
*Puccinia pulverulenta* NZFRI 29019	KM065015	–
*Puccinia rapipes* Prap_4	MK874620	–
*Puccinia rhagodiae* BRIP 60078	–	KX999890
*Puccinia saccardoi* BRIP 57725	–	KF690701
*Puccinia salihae* BPI 881123	–	HQ412645
*Puccinia setariae* BPI 188745	NR148110	–
*Puccinia smilacis* BPI 871784	DQ354533	–
*Puccinia sporoboli* var. *robusta* BPI 871549	–	GU058003
*Puccinia striiformoides* BPI 199096	HM057137	–
*Puccinia suaveolens* KRM 0005945	ON063373	–
*Puccinia taeniatheri* IRAN 11491	AY956557	–
*Puccinia tuberosa* HMJAU 8949	OK489429	OK489421
*Puccinia ursiniae* BRIP 57993	–	–
*Puccinia violae* BPI 842321	DQ354509	–
*Puccinia wiehei* BPI 111530	NR148111	–
*Puccinia windhoekensis* NA 152	–	DQ917710
*Puccinia xanthii* BRIP 48819	EU659694	–
*Puccinia xanthosiae* BRIP 57729	–	KF690706
*Puccinia xinyuanensis* HMUT 2549	NR173762	NG079639
*Uromyces scaevolae* BRIP 60096	–	KJ622214

**Table 2:** Decadal and centurial trends of published literature on *Puccinia*.

Year Range	Number of Publications	
	Decadal Trend	Centurial Trend
1794–1800	02	02
1801–1810	03	277
1811–1820	06	
1821–1830	09	
1831–1840	06	
1841–1850	03	
1851–1860	09	
1861–1870	09	
1871–1880	61	
1881–1890	50	
1891–1900	121	
1901–1910	144	627
1911–1920	85	
1921–1930	51	
1931–1940	71	
1941–1950	58	
1951–1960	78	
1961–1970	28	
1971–1980	44	
1981–1990	39	
1991–2000	29	
2001–2010	42	82
2011–2022	40	

**Table 3:** Species of *Puccinia* that cause destructive diseases in agricultural and nonagricultural crops.

Rust Disease	Causal Organism	Host Plant	Reference
Rust disease of eucalyptus	*Puccinia psidii*	*Eucalyptus* spp.	[46]
Stripe rust of wheat	*Puccinia striiformis*	*Triticum* sp.	[38,59]
Stem rust of wheat	*Puccinia graminis*	*Triticum* sp.	[36,60]
Guava rust	*Puccinia psidii*	*Psidium guajava*	[48]
Crown rust of cultivated and wild oats	*Puccinia coronata* f.sp. *avenae*	*Avena sativa*	[44]
White rust of chrysanthemum	*Puccinia horiana*	*Chrysanthemum* sp.	[61]
Geranium rust	*Puccinia pelargonic-zonalis*	*Pelargonium × hortorum*	[62]
Viola rust	*Puccinia viola* and *P. pulchella*	*Viola* sp.	[63]
Garlic rust	*Puccinia allii*	*Allium sativum*	[64]
Switchgrass rust	*Puccinia* *emaculata*	*Panicum* *virgatum*	[65]
Senecio rust	*Puccinia lagenophorae*	*Senecio vulgaris*	[66]
Peanut rust	*Puccinia arachidis*	*Arachis* spp.	[67]
Oxalis rust	*Puccinia oxalidis*	*Oxalis latifolia*	[68,69]
Buckwheat rust	*Puccinia fagopyri*	*Fagopyrum esculentum*	[54]
Mint rust	*Puccinia menthae*	*Mentha longifolia*	[54]

## Data Availability

Not applicable.
